# Meteorological Register for June, 1817

**Published:** 1817-08

**Authors:** John Bailey

**Affiliations:** Surgeon, &c.


### Meteorological Register for June, 1817,

*Kept at Harwich, in Essex,*

By JOHN BAILEY, Surgeon, &c.

| Moon's Age. | | Atmospherical Preſſure. Morn. | Ev. | Temp. | Wind. | Remarks. | General Statement of Diſeaſes. |
|---|---|---|---|---|---|---|---|
| | 1 | 29 7 | 29 7 | 61 | W | Dull and Cloudy | |
| | 2 | 7 | 7 | 64 | W | Rain | Intermittentes. |
| | 3 | 5½ | 6½ | 64 | W | Rain. Brisk Gale. | |
| | 4 | 6½ | 8 | 63 | W | Ditto Violent Gale | Parotidea |
| | 5 | 30 | 30 | 64 | W | | |
| ☽ | 6 | 30 | 30 | 67 | W | Fine and clear | Cholera |
| | 7 | 29 9 | 29 7½ | 72 | SE | Ditto | |
| | 8 | 6 | 7½ | 68 | W | Cloudy. Rain | |
| | 9 | 8 | 7½ | 62 | W | Much Rain | Rheumatismus |
| | 10 | 7 | 8½ | 64 | NW | ditto | Acutus. |
| | 11 | 9 | 8 | 60 | W | Fine | |
| | 12 | 7½ | 7 | 64 | W | Rain. Cloudy | Pneumonia |
| | 13 | 6 | 5 | 64 | W | Much Rain | |
| ● | 14 | 5 | | 50 | SW | Gale of Wind | |
| | 15 | 30 | 30 1 | 62 | | Fine and clear | |
| | 16 | 2 | 2 | 60 | SE | Ditto | |
| | 17 | 1 | 29 8 | 65 | SE | | |
| | 18 | 29 7½ | 7 | 67 | SE | | |
| | 19 | 6½ | 7 | 72 | SE | | |
| | 20 | 7½ | 7½ | 75 | E | | |
| ☽ | 21 | 8 | 8½ | 80 | E | | |
| | 22 | 9 | 9 | 76 | E | | |
| | 23 | 9 | 9 | 79 | E | | |
| | 24 | 8½ | 8½ | 79 | W | | |
| | 25 | 8½ | 8½ | 80 | S | | |
| | 26 | 8 | 6½ | 72 | S | | |
| | 27 | 6 | 5 | 69 | E | Showery & Dull | |
| ○ | 28 | 5½ | 8 | 69 | W | Brisk Wind | |
| | 29 | 8½ | 7½ | 68 | SE | Dull and Cloudy | |
| | 30 | 6½ | 7½ | 68 | W | Rain | |

